# Poor Glycemic Control and Its Predictors Among Type 2 Diabetes Patients: Insights From a Single‐Centre Retrospective Study in Ghana

**DOI:** 10.1002/hsr2.70558

**Published:** 2025-03-11

**Authors:** Samuel Kyeremeh Adjei, Prosper Adjei, Patience Adasah Nkrumah

**Affiliations:** ^1^ Department of Internal Medicine Methodist Hospital Wenchi Ghana; ^2^ School of Public Health and Allied Sciences Catholic University of Ghana Fiapre‐Sunyani Ghana

**Keywords:** Diabetes mellitus, Ghana, Glycemic control, Glycosylated hemoglobin

## Abstract

**Background and Aims:**

The primary objective of glycemic control in individuals with diabetes mellitus is to avert or postpone complications, which ultimately leads to an improved quality of life. Nonetheless, achieving the recommended targets for glycemic control in clinical settings often proves challenging. Consequently, it is crucial to ascertain factors that affect glycemic outcomes to enhance the management of diabetes mellitus. This study sought to evaluate the levels of glycemic control and the associated factors among patients with type 2 diabetes receiving care at the Methodist Hospital, Wenchi, Ghana.

**Methods:**

A retrospective study was conducted using an existing database. Glycemic control was evaluated by HbA1c measurements with a target of < 7% indicating good control, as per the guidelines established by the American Diabetes Association for nonpregnant adults. HbA1c levels ≥ 7% were classified as poor control. Data analysis was conducted using SPSS version 25 and multivariate logistic regression analysis was employed to determine the factors affecting glycemic control.

**Results:**

The median HbA1c level among the participants was 7.9% (IQR: 5.8–9.9). Majority (59.3%) demonstrated poor glycemic control with HbA1c ≥ 7%. Factors associated with poor glycemic control included advanced age (AOR: 4.32, 95% CI: 0.61–11.21, *p* = 0.012), duration of diabetes mellitus > 10 years (AOR: 3.60, 95% CI: 1.05–9.82, *p* = 0.019), insulin therapy (AOR: 3.13, 95% CI: 0.55–11.01, *p* = 0.009) and hypertension diagnosis (AOR: 2.88, 95% CI: 0.75–5.45, *p* = 0.030).

**Conclusions:**

The study indicated that a considerable proportion of individuals with diabetes exhibited inadequate glycemic control. Older age, longer duration of diabetes mellitus, insulin therapy and comorbid hypertension were significantly associated with poor glycemic control among the study population. Multidisciplinary interventions as well as customized management strategies are required to ensure effective glycemic control to prevent long‐term complications of diabetes mellitus.

**Abbreviations**
AOR: adjusted odds ratio
BMI: body mass index
CI: confidence interval
COR: crude odds ratio
HbA1c: glycosylated hemoglobin
IDF: international diabetes federation
SD: standard deviation
SMD: standardized mean differences
T2DM: type 2 diabetes mellitus

## 1 Introduction

Diabetes mellitus is a metabolic disorder marked by persistent hyperglycemia which arises from inadequate utilization of glucose for energy and its excessive production due to dysregulated glucose metabolism including gluconeogenesis and glycogenolysis [1]. Type 2 diabetes mellitus is the most prevalent type of diabetes, comprising 90% of all diagnosed cases while type 1 diabetes and gestational diabetes account for the remaining 10% [2].

Diabetes mellitus represents a major global health concern that transgresses territorial borders, impacting individuals in both high and low‐income countries [3]. In 2021, it was estimated that approximately 537 million people aged 20–79 worldwide were diagnosed with diabetes, leading to around 6.7 million deaths linked to the condition [4]. The International Diabetes Federation (IDF) indicated that in 2021, around 24 million adults aged 20–79 were identified as having diabetes within the IDF African Region, indicating a regional prevalence rate of 4.5% and resulted in 416,000 fatalities. Furthermore, the report indicated that around 329,000 adults in Ghana were living with diabetes in the same year, reflecting a prevalence of 2.0% [5].

Effective glycemic control is crucial for the management of diabetes. Glycosylated hemoglobin (HbA1c) which serves as an indicator of the average plasma glucose concentration over the past 3 months [6], is a reliable measure of glycemic control. Nonetheless, worldwide data demonstrate that HbA1c levels are often suboptimal, especially in low‐ and middle‐income countries [7, 8, 9, 10, 11, 12]. Studies conducted in Ghana indicate that the rate of insufficient glycemic control surpasses 59%, as indicated by HbA1c levels of ≥ 7% [13, 14].

The devastating effects of inadequate glycemic regulation include macrovascular and microvascular complications such as retinopathy, nephropathy, cerebrovascular events and ischemic heart disease [15]. Elevated HbA1c correlates significantly with the risk of developing long‐term complications of diabetes mellitus [16].

The findings from the United Kingdom Prospective Diabetes Study (UKPDS) indicate that a 1% decrease in HbA1c% correlates with a 21% decrease in mortality related to diabetes, a 14% lower risk of myocardial infarction and a 37% reduction in the incidence of microvascular complications [17]. However, studies reveal that fewer than 50% of individuals with diabetes attain proper glycemic control as reflected in their HbA1c levels [18].

Numerous factors have been identified as impacting glycemic control in individuals with diabetes. Recognizing these factors is crucial for creating interventions that enhance glycemic control and mitigate the risk of target organ damage as well as other long‐term complications associated with diabetes. This study sought to evaluate glycemic control levels in patients with type 2 diabetes and to determine the factors that independently affect these levels.

## 2 Methods

### 2.1 Study Design, Setting and Period

A retrospective analysis was performed using pre‐existing medical records from the hypertension and diabetes clinic at the Methodist Hospital, Wenchi, Ghana. This study was carried out over a period extending from April 2022 to March 2023. The hypertension and diabetes clinic at the Methodist Hospital, Wenchi offers extensive healthcare services to a significant proportion of patients with diabetes and hypertension in the Wenchi Municipality, located in the Bono region of Ghana. Approximately 1,200 patients seek medical care at the clinic annually. Ethical approval and a waiver for informed consent were granted by the Research Committee of Methodist Hospital, Wenchi (Reference No. MHW/MPD/104).

### 2.2 Study Population, Inclusion and Exclusion Criteria

The study involved individuals aged ≥ 18 years who had been diagnosed with type 2 diabetes and had been receiving treatment at the facility for a minimum of six continuous months. Individuals with type 1 diabetes, gestational diabetes or secondary diabetes mellitus were not included in the study.

### 2.3 Study Variables

The primary outcome measure was the degree of glycemic control assessed through the estimation of HbA1c levels utilizing the SD Biosensor F200 Analyzer (SD Biosensor Inc. Republic of Korea). The predictor variables included sociodemographic characteristics such as age, gender, marital status, educational attainment, occupation, place of residence, health insurance status, smoking habits and alcohol consumption, as well as clinical factors including family history of diabetes, duration of diabetes, body mass index (BMI), treatment regimen and the presence of hypertension.

### 2.4 Statistical Analysis

Data analysis was conducted using SPSS version 25. To summarize the baseline sociodemographic characteristics, descriptive statistics such as frequencies, percentages and median were employed. Furthermore, logistic regression analysis was employed to ascertain the determinants of poor glycemic control. Variables that exhibited a *p*‐value < 0.05 in the bivariate analysis were subsequently included in the multivariate logistic regression analysis. This multivariate analysis aimed to identify independent predictors associated with poor glycemic control. Statistically significant relationships were established based on the adjusted odds ratio (AOR), its 95% confidence interval (CI) and a *p*‐value < 0.05. A propensity score matching (PSM) approach was employed to evaluate the effect of treatment type (insulin therapy vs. oral medications) on glycemic control among type 2 diabetes mellitus patients. This analysis aimed to reduce confounding bias caused by baseline differences in patient characteristics such as age, BMI, duration of diabetes and hypertension which may influence both treatment choices and glycemic control. Propensity scores were calculated through a logistic regression analysis, where the treatment type served as the dependent variable and the identified confounding factors were treated as independent variables. Matching was performed using 1:1 nearest‐neighbor matching without replacement, applying a caliper of 0.2 standard deviations of the logit of the propensity score to ensure close matches. The effectiveness of matching was evaluated by assessing the standardized mean differences (SMD) for each covariate. An SMD < 0.1 was considered indicative of adequate balance.

### 2.5 Operational Definitions

#### 2.5.1 Good Glycemic Control

HbA1c value < 7% [19].

#### 2.5.2 Poor Glycemic Control

HbA1c value ≥ 7% [19].

## 3 Results

### 3.1 Sociodemographic Characteristics of the Participants

The study consisted of 248 patients, out of which 30.2% (*n* = 75) were males and 69.8% (*n* = 173) were females. The average age of the participants was 57.6 years (SD ± 10.9) and more than one‐third (35.9%) of them were in the 55–64 age group. Notably, 73% (*n* = 181) of the patients were married and 33.9% (*n* = 84) of them also lacked formal education. In terms of employment status, 64.5% (*n* = 160) were self‐employed while 18.5% (*n* = 46) were employed by government or private institutions. Most (67.3%) of them were urban dwellers. A substantial majority of the participants (95.2%) were actively enrolled on various Health Insurance Schemes. Furthermore, the majority of participants were categorized as non‐smokers and nondrinkers [Table 1].

**Table 1:** Sociodemographic characteristics of participants with T2DM, MHW, Ghana, 2024.

Characteristic	Frequency	Percentage (%)
**Age (in years)**		
Age (mean)	57.6 (SD ± 10.9)	
18–44	28	11.3
45–54	73	29.4
55–64	89	35.9
≥ 65	58	23.4
**Gender**		
Male	75	30.2
Female	173	69.8
**Marital status**		
Single	16	6.5
Married	181	73.0
Divorced	10	4.0
Widow(er)	41	16.5
**Educational status**		
No formal education	84	33.9
Primary	65	26.2
Secondary	69	27.8
Tertiary	30	12.1
**Occupation**		
Unemployed	40	16.1
Self‐employed	160	64.5
Gov't/Private employee	46	18.5
Retired/Pension	2	0.8
**Residence**		
Urban	167	67.3
Rural	81	32.7
**Health Insurance**		
Yes	236	95.2
No	12	4.8
**Smoking**		
No	243	98.0
Yes	5	2.0
**Alcohol**		
No	244	98.4
Yes	4	1.6

### 3.2 Clinical Characteristics of Participants With Type 2 Diabetes Mellitus

A family history of diabetes mellitus was noted in 14.9% (*n* = 37) of the individuals surveyed. The median duration since diabetes diagnosis among participants was 4 years (IQR: 2.0–6.0). Seven percent of participants had been living with diabetes for > 10 years. The majority (52.4%) of individuals had a normal BMI while 30.2% (*n* = 75) were categorized as overweight. Additionally, 31 participants (12.5%) were classified as obese and 4.8% were identified as underweight. The majority of the study participants (76.6%) were on oral hypoglycemic medications while 4.0% (*n* = 10) used insulin. Furthermore, 48 (19.4%) participants utilized a combination of both insulin and oral hypoglycemic agents. Comorbid conditions, particularly hypertension was reported in 62.5% (*n* = 155) of the patients [Table 2].

**Table 2:** Glycemic control and clinical characteristics of participants with T2DM, MHW, Ghana, 2024.

Characteristic	Frequency	Percentage (%)
**Family history of T2DM**		
Yes	37	14.9
No	211	85.1
**Duration of T2DM**		
DM duration, median (IQR)	4.0 (2.0–6.0)	
< 2 years	52	21.0
2–5 years	142	57.3
6–10 years	36	14.5
> 10 years	18	7.3
**BMI (kg/m** ^**2**^ **)**		
Under weight (< 18.5)	12	4.8
Normal (18.5–24.9)	130	52.4
Over weight (25–29.9)	75	30.2
Obese (> 30)	31	12.5
**Therapeutic regimen**		
Oral hypoglycemic agents	190	76.6
Insulin	10	4.0
Combination of both	48	19.4
**Hypertension**		
Yes	155	62.5
No	93	37.5
**HbA1c (%)**		
HbA1c, median (IQR)	7.9 (5.8–9.9)	
< 7	101	40.7
≥ 7	147	59.3

Abbreviations: BMI, Body Mass Index; HBA1c, hemoglobin A1c; MHW, Methodist Hospital, Wenchi; T2DM, Type 2 diabetes mellitus.

### 3.3 Glycemic Control Among Participants

The median HbA1c level among the participants in the study was 7.9% (IQR: 5.8–9.9). It was found that 101 (40.7%) individuals had good glycemic control (HbA1c < 7%). A significant majority (59.3%) demonstrated poor glycemic control (HbA1c ≥ 7%) [Table 2].

### 3.4 Impact of Treatment Type on Glycemic Control

A total of 248 T2DM patients were incorporated in the initial propensity score matching analysis, with 120 patients (60 per group) retained after 1:1 nearest‐neighbor matching. Before matching, substantial differences were observed between the treatment groups for key covariates, as indicated by standardized mean differences (SMD) exceeding 0.1. After matching, all covariates achieved balance with SMD values below 0.1 confirming the success of the matching process in reducing baseline differences (Table 3).

**Table 3:** Covariate balance before and after matching.

Covariates	SMD (before matching)	SMD (after matching)
Age	0.45	0.02
BMI	0.38	0.05
Gender	0.12	0.02
Duration of diabetes	0.51	0.04
Hypertension	0.20	0.01

Abbreviation: SMD, standardized mean differences.

After matching, the prevalence of poor glycemic control (HbA1c ≥ 7%) was remarkably greater among the insulin therapy group (75%, *n* = 45) compared to the oral medication group (60%, *n* = 36). Conversely, good glycemic control (HbA1c < 7%) was observed in 25% (*n* = 15) of the insulin group and 40% (*n* = 24) of the oral medication group. Statistical comparison using McNemar's test for paired data revealed a significant difference in glycemic control outcomes between the two groups (Chi‐square = 4.64, *p*‐value = 0.03) [Table 4].

**Table 4:** Comparison of glycemic control between insulin and oral medication groups post‐matching.

Group	Poor glycemic control (*n*, %)	Good glycemic control (*n*, %)
Insulin therapy	45 (75)	15 (25)
Oral medications	36 (60)	24 (40)

### 3.5 Factors Associated With Poor Glycemic Control

In the bivariate logistic regression analysis, factors such as age, gender, marital status, occupation, duration since diabetes diagnosis, BMI, therapeutic regimen and the presence of hypertension were aligned with poor glycemic control.

The findings from the subsequent multivariate logistic regression analysis indicated that several variables were significantly associated with poor glycemic control. These included advanced age (AOR: 4.32, 95% CI: 0.61–11.21, *p* = 0.012), duration of diabetes mellitus > 10 years (AOR: 3.60, 95% CI: 1.05–9.82, *p* = 0.019), insulin therapy (AOR: 3.13, 95% CI: 0.55–11.01, *p* = 0.009) and the presence of hypertension (AOR: 2.88, 95% CI: 0.75–5.45, *p* = 0.030) [Table 5].

**Table 5:** Bivariate and multivariate logistic regression analysis of factors associated with poor glycemic control among T2DM patients at MHW, Ghana, 2024.

Characteristic	Glycemic control (HbA1c)		Bivariate analysis		Multivariate	
	Good	Poor	COR (95% CI)	*p* value	AOR (95% CI)	*p* value
	(*n* = 101)	(*n* = 147)				
**Age**						
18–44	10 (35.7)	18 (64.3)	1		1	
45–54	27 (37.5)	46 (63.0)	0.81 (0.38–2.35)	0.123	1.41 (0.50–3.94)	0.518
55–64	39 (43.8)	50 (56.2)	2.16 (0.29–1.72)	0.009*	4.32 (0.61–11.21)	0.012*
≥ 65	25 (43.1)	33 (58)	0.63 (0.28–1.86)	0.450	2.10 (1.92–5.28)	0.021*
**Duration of T2DM**						
< 2 years	21 (40.4)	31 (59.6)	1		1	
2–5 years	54 (38.0)	88 (62.0)	0.74 (0.24–2.32)	0.597	1.98 (0.23–4.18)	0.205
6–10 years	20 (55.6)	16 (44.4)	0.95 (0.29–2.64)	0.039*	2.17 (0.45–7.02)	0.802
> 10 years	6 (33.3)	12 (66.7)	2.38 (0.32–5.67)	0.026*	3.60 (1.05–9.82)	0.019*
**Therapeutic regimen**						
Oral hypoglycemic agents	76 (40.0)	114 (60.0)	1			
Insulin	5 (50.0)	5 (50.0)	2.57 (0.56–9.10)	0.004*	3.13 (0.55–11.01)	0.009*
Combination of both	20 (41.7)	28 (58.3)	1.96 (0.27–4.82)	0.833	1.43 (0.36–4.21)	0.604
**Hypertension**						
No	44 (47.3)	49 (52.7)	1		1	
Yes	57 (36.8)	98 (63.2)	2.12 (0.65–6.21)	0.015*	2.88 (0.75–5.45)	0.030*

Abbreviations: AOR, adjusted odds ratio; CI, confidence interval; COR, crude odds ratio; HbA1c, Hemoglobin A1c; T2DM, Type 2 diabetes mellitus.

* Statistically significant.

## 4 Discussion

The primary therapeutic objective in diabetes management is to achieve good glycemic control, which is essential for preventing organ damage and associated complications [20]. HbA1c is an important and a reliable measure of glycemic control. This study revealed that a significant proportion of participants, specifically 59.3%, exhibited inadequate glycemic control (HbA1c ≥ 7%). This is comparable to studies conducted in Ghana (61.2%) [14] and Ethiopia (61.1%) [21]. The extent of suboptimal glycemic control observed in the present study exceeded that documented in prior studies in India (37.5%) [22], Tanzania (49.8%) [23] and Ethiopia (45.2%) [24]. This disparity may be ascribed to the variations in study environment, genetic factors and dietary differences. However, the prevalence of poor glycemic control reported in this study is lower compared to several earlier studies in Egypt (92.3%) [25], Saudi Arabia (74.9%) [26], Ghana (70%) [27], Uganda (73.5%) [28], Ethiopia (72.8%) [29], Nigeria (83.3%) [30] and Kenya (81.6%) [31]. The variation observed between the current and earlier studies conducted in sub‐Saharan Africa could be due to differences in methodologies employed in determining glycemic control. Some studies relied on fasting blood sugar (FBS) assessments while others utilized HbA1c measurements. Another consideration is the application of various assay techniques, particularly regarding the measurement of HbA1c. The use of assays that are not certified by the National Glycohemoglobin Standardization Program (NGSP) and not standardized according to the Diabetes Control and Complications Trial (DCCT), can result in inaccurately elevated or reduced values [32]. Additionally, the recorded variation may be attributed to the clinical and sociodemographic characteristics of the study participants.

Factors such as advanced age, duration of diabetes mellitus > 10 years, insulin therapy and the presence of comorbidities, particularly hypertension, were strongly linked to poor glycemic control. The study found that individuals aged ≥ 55 years with diabetes were more prone to suboptimal glycemic control compared to younger individuals. This observation aligns with the outcomes reported in earlier studies [30, 33]. This phenomenon can be partially attributed to the more lenient glycemic targets established for older adult patients, taking into account factors such as reduced life expectancy, the presence of multiple comorbidities and significant microvascular or macrovascular complications, where the associated risks and burdens surpass the possible advantages of rigorous glycemic management.

Individuals diagnosed with diabetes mellitus for a duration > 10 years were 3.60 times more likely to have poor glycemic control compared to those with a shorter disease duration. This observation is supported by previous studies [34, 35, 36]. The chronic and progressive nature of diabetes mellitus can lead to challenges in achieving optimal glycemic control for patients who have had the disease for an extended period. This difficulty may be attributed to impaired insulin secretion resulting from dysfunction of pancreatic beta cells [37].

Treatment modality has a considerable impact on the outcomes of glycemic control. None of the participants in this study utilized insulin pumps or continuous glucose monitoring systems. Individuals undergoing insulin therapy exhibited a 3.13‐fold increased likelihood of experiencing inadequate glycemic control compared to those on oral hypoglycemic agents. This finding is consistent with the outcomes reported in other studies [12, 28, 38]. Factors that could contribute to inadequate glycemic control among individuals utilizing insulin include injection phobia or the overall inconvenience associated with the use of insulin, inappropriate dosages and poor storage conditions of insulin that decreases its potency. Moreover, the findings of this study reveal that, even after adjusting for potential confounders through propensity score matching analysis, insulin therapy was associated with a higher likelihood of poor glycemic control juxtaposed with oral medications. This may be partly explained by the fact that insulin therapy is often prescribed to patients with more advanced disease or those who fail to achieve adequate glycemic control with oral medications alone. Additionally, insulin therapy requires substantial patient education and adherence to complex dosing regimens, which may be challenging for many patients. Issues such as fear of hypoglycemia, difficulty with self‐monitoring of blood glucose and inadequate support systems may result in suboptimal insulin use, thereby contributing to poor glycemic control. In contrast, oral medications, which are often easier to administer and require less patient effort, may facilitate better adherence and consequently improve glycemic outcomes in selected patients. In low‐resource settings like Ghana, constraints such as high out‐of‐pocket costs and inadequate patient support systems may disproportionately affect insulin‐treated patients. These systemic barriers may explain the poorer outcomes observed in this group despite the theoretical superiority of insulin in glycemic control. These observations highlight the necessity for focussed actions such as improved patient education and support to enhance glycemic control outcomes in patients requiring insulin therapy.

Additionally, individuals with a diagnosis of hypertension as a comorbid condition exhibited a 2.88‐fold increased likelihood of experiencing poor glycemic control in comparison to those with normal blood pressure. This observation aligns with a study conducted in Malaysia [39]. The possible reasons for this observation could be the pathophysiological interplay between diabetes and hypertension that results in increased insulin resistance and the interactions between medications used for managing both conditions. It is worth noting that, the coexistence of hypertension and diabetes mellitus significantly accelerates the development and progression of diabetes‐related complications. Evidence from several studies indicate that poorly controlled hypertension in individuals with diabetes mellitus can result in the onset and exacerbation of diabetic retinopathy [40]. Also, uncontrolled hypertension contributes greatly to the development and rapid progression of diabetic nephropathy [41]. Hypertension again, has been found to be a major modifiable risk factor for diabetic neuropathy, particularly distal symmetrical polyneuropathy [42]. In view of the foregoing, it is extremely important for clinicians to ensure adequate glycemic and blood pressure control in individuals with concurrent diabetes mellitus and hypertension to prevent or slow the progression of these devastating complications of diabetes mellitus.

The study presents several advantages and limitations. It is the first study ever to be carried out to ascertain the determinants of poor glycemic control among type 2 diabetics receiving medical care at the Methodist Hospital, Wenchi, Ghana. Regarding generalizability, the insights from this study may hold significance for contexts that feature comparable demographic characteristics and healthcare infrastructures.

The absence of a significant association between educational level and glycemic control in our study warrants careful consideration, particularly in light of well‐documented relationship between socioeconomic status and diabetes in existing literature [43, 44, 45]. The small sample size of this current study may have influenced this outcome. While our study was powered to detect differences in broader diabetes‐related parameters, it may not have been adequately powered to identify associations between educational level and glycemic control. Larger and more diverse samples may be required to replicate findings from previous studies and detect such relationships. Additionally, it is worth considering that the interplay of other variables, such as duration of diabetes, therapeutic regimen and presence of comorbid hypertension may have attenuated the direct effect of educational level on glycemic control within our study. Previous studies have suggested that while educational level is an important determinant of health, its effects may be influenced or obscured by other variables [46, 47].

This retrospective analysis was performed using a pre‐existing database and data quality issues such as accuracy cannot be ignored. Certain data contained in the medical records of the participants were obtained through self‐reporting (e.g., duration of type 2 diabetes mellitus), which may have introduced recall bias.

## 5 Conclusion

The study unveiled that most individuals (59.3%) exhibited inadequate glycemic control contrary to the recommended HbA1c target of < 7%. Notable links to inadequate glycemic control were found in connection with older age, a longer duration of diabetes mellitus, insulin therapy and diagnosis of hypertension. This underscores the urgent need for multidisciplinary and comprehensive interventions aimed at addressing these factors to enhance glycemic control in type 2 diabetics. Frequent follow‐up appointments to evaluate and monitor individuals with the aforementioned factors may be required.

Additionally, it is recommended that more rigorous glycemic targets (HbA1c < 6.5%) be set for patients with diabetes mellitus who have a relatively short disease duration and a long‐life expectancy provided these targets can be met safely without causing significant hypoglycemic events. Conversely, more lenient targets (HbA1c up to 8%) are advised for individuals with a limited life expectancy, notable comorbid conditions, progressive microvascular or macrovascular complications and those who experience recurrent or grave hypoglycemic events. A comprehensive longitudinal study is necessary to evaluate the influence of multiple factors including those mentioned on the glycemic control of these patients over an extended period.

## Author Contributions

**Samuel Kyeremeh Adjei:** conceptualization, formal analysis, writing – original draft, writing – review and editing, methodology. **Prosper Adjei:** writing – review and editing. **Patience Adasah Nkrumah:** data curation.

## Ethics Statement

Approval was obtained from the Research Committee of the Methodist Hospital, Wenchi (Ref No. MHW/MPD/104) before the commencement of the study. The study did not incorporate any identifying details such as patient name or identification number, ensuring that all data was handled with the highest level of confidentiality. Additionally, the requirement for informed consent was waived by the Research Committee.

## Conflicts of Interest

The authors declared no potential conflicts of interest with respect to the authorship and/or publication of this article.

## Transparency Statement

The lead author Samuel Kyeremeh Adjei affirms that this manuscript is an honest, accurate, and transparent account of the study being reported; that no important aspects of the study have been omitted; and that any discrepancies from the study as planned (and, if relevant, registered) have been explained.

## Data Availability

The datasets used and/or analyzed during this study are available from the corresponding author upon reasonable request.
